# Temporal improvements in loco-regional failure and survival in patients with anal cancer treated with chemo-radiotherapy: treatment cohort study (1990–2014)

**DOI:** 10.1038/s41416-019-0689-x

**Published:** 2020-01-14

**Authors:** Hema Sekhar, Lee Malcomson, Rohit Kochhar, Matthew Sperrin, Nooreen Alam, Bipasha Chakrbarty, Paul E. Fulford, Malcolm S. Wilson, Sarah T. O’Dwyer, Mark P. Saunders, Andrew G. Renehan

**Affiliations:** 1 Division of Cancer Sciences, School of Medical Sciences, Faculty of Biological, Medicine and Health, University of Manchester, Manchester, UK; 2 Department of Radiology, The Christie NHS Foundation Trust, Manchester, UK; 3 Health eResearch Centre, Farr Institute, University of Manchester, Manchester, UK; 4 Department of Clinical Oncology, The Christie NHS Foundation Trust, Manchester, UK; 5 Department of Pathology, The Christie NHS Foundation Trust, Manchester, UK; 6 Colorectal and Peritoneal Oncology Centre, The Christie NHS Foundation Trust, Manchester, UK

**Keywords:** Anal cancer, Cancer therapy

## Abstract

**Background:**

We evaluated oncological changes in patients with squamous cell carcinoma of the anus (SCCA) treated by chemoradiotherapy (CRT) from a large UK institute, to derive estimates of contemporary outcomes.

**Methods:**

We performed a treatment-cohort analysis in 560 patients with non-metastatic SCCA treated with CRT over 25 years. The primary outcomes were 3-year loco-regional failure (LRF), 5-year overall survival (OS), and 5-year cancer-specific survival (CSS). We developed prediction models; and overlaid estimates on published results from historic trials.

**Results:**

Age distributions, proportions by gender and cT stage remained stable over time. The median follow-up was 61 (IQR: 36–79) months. Comparing the first period (1990–1994) with the last period (2010–2014), 3-year LRF declined from 33 to 16% (*P*_trends_ < 0.001); 5-year OS increased from 60% to 76% (*P*_trends_ = 0.001); and 5-year CCS increased from 62% in to 80% (*P*_trends_ = 0.001). For 2020, the models predicted a 3-year LRF of 14.7% (95% CIs: 0–31.3); 5-year OS of 74.7% (95% CIs: 54.6–94.9); and 5-year CSS of 85.7% (95% CIs: 75.3–96.0). Reported oncological outcomes from historic trials generally underestimated contemporary outcomes.

**Conclusions:**

Current and predicted rates for 3-year LRF and 5-year survivals are considerably improved compared with those in historic trials.

## Background

Large-scale population-based studies in developed countries, such as EUROCARE,^1^ indicate that ‘major advances in cancer management seem to have resulted in improved survival’ in many cancer types. These data are informative for policy-makers seeking information on net survival improvements but generally lag behind contemporary management strategies (for example, EUROCARE reports only to 2007^1^); focus mainly on common cancers; and generally fail to capture detailed treatment and stage information, necessary to interpret whether survival improvements reflect introductions of new treatments or stage migrations.

In contrast, patients and oncologists generally seek to understand prognosis, namely the chance of surviving from a specific cancer, in the context of contemporary treatment options.^2^ For trialists, there is an additional need to forecast expected number of events based on current standard of care. But a new problem is emerging in trials—namely that outcomes from contemporary standard arm management exceed expectations (compared with historical literature). Thus, trials reach target recruitment but findings appear to lack power.^3^ This issue is exemplified in recent non-oncology (ARRIVE^4^) and oncology (COLOFOL^5^ and ROLAAR^6^) trials.

Here, we address the above problem in the setting of an uncommon cancer, namely squamous cell carcinoma cancer of the anus (SCCA), treated with chemo-radiotherapy (CRT). The latter is standard of care in many countries as reflected by guidelines, for example, from NCCN,^7^ ESMO-ESSO-ESTRO,^8^ and ACPGBI.^9^ Approximately three-quarters of patients with SCCA receive CRT as initial treatment.^10^ Through systematic review,^11^ we recently reported on 45 studies of patients with SCCA who received either radiotherapy alone (RT) or CRT and noted that 5-year overall survival increased from a mean estimate of 64% in 1980 to 75% in 2010 (*p* = 0.046). It is conceivable that this temporal improvement might be driven by improvements in loco-regional control, but might also be due to unmeasured factors, such as general improvement in healthcare, centralisation, improved imaging and radiotherapy delivery, and more effective management of toxicity. It might also reflect early tumour stage at presentation or younger mean age at diagnosis.

In this study, we confirmed the observation of significant temporal improvement in survivals and aimed to use these striking temporal changes to derive models to estimate contemporary outcomes.

## Methods

### Patients

We performed a treatment-cohort analysis, using a prospectively maintained clinical database of patients with SCCA treated at the Christie NHS Foundation Trust, Manchester, United Kingdom, seen between 1 January 1990 and 31 December 2014, and followed to 30 April 2018. The Christie anal cancer multi-disciplinary team (MDT) meeting was centralised for the Greater Manchester and North Cheshire geographical areas (approximate 1.8 million) in 2007. From 2004, pre-treatment HIV testing was performed selectively (for example, untested male homosexual men).

Patients were included if they had histologically confirmed squamous cell carcinoma arising from the anal canal or margin treated with CRT with curative intent. For sensitivity analyses, patients treated curatively with RT alone were added. Standard clinical, pathological and treatment-related variables were collected, as previously published.^12^ We recognised a change in pre-treatment staging assessment through the study period and categorised this as follows: 1990 to 2003 assessment was physical examination and CT imaging; 2004 to 2010 assessment added MR imaging;^13^ and from 2011 to 2014, assessment additionally added Fluoro-Deoxy-Glucose Positron Emission Tomography/Computed Tomography. TNM staging was in accordance with the American Joint Committee on Cancer (AJCC) staging 7th Edition.^14^

### Treatment

From 1990 to 2001, a split ACT I^15^ radiotherapy regimen was prescribed and described elsewhere.^12^ After 2001, the treatment protocol followed that used in the ACT II trial^16^—namely, radiotherapy of 50.4 Gy was delivered over 5.5 weeks with a two phase technique, without a mandatory break. Phase 1 included 30.6 Gy in 17 daily fractions with non-conformal rectangular parallel-opposed fields. Phase 2 required conformal planning and delivered 19.8 Gy in 11 daily fractions over 15 days to the primary tumour with a 3 cm margin and any involved lymph nodes. From 2005, we reported median duration of radiotherapy treatment.

Chemotherapy regimens were administered concurrently with radiotherapy as either: mitomycin-C (MMC) 12 mg/m^2^ on day 1, and continuous infusion of 5-fluorouracil (5-FU) 1000 mg/m^2^ on days 1–4 and days 29–32 or cisplatin (60 mg/m^2^ on days 1 and 29) with 5-FU (as above), the latter regimen as part of the ACT II trial^16^ (2001–2008). The selection to RT or CRT was randomized as part of the ACT I trial^15^ until 1994. Thereafter, selection for RT was the exception, and based on contra-indications to the use of CRT, typically co-morbidities or increasing age.

### Follow-up and outcomes

Since 2004, post-treatment follow-up was typically clinical assessment at 6 weeks after completion of CRT and again at clinical visits paralleling the 3- and 6-month MR scans.^13^ From 6 to 60 months, patients were assessed clinically on a six-monthly basis and imaging follow-up based on risk of local relapse—in patients deemed at high-risk for local relapse (T size > 5 cm; AJCC 7th Edition N2 and N3 disease; incomplete RT or CRT), MR scans were generally performed at 12, 18, 24 and 36 months; in the remainder (low-risk), MR scans were performed at 36 months. Prior to 2004, surveillance was by clinical examination.

For this analysis, the primary outcomes were 3-year loco-regional failure (LRF); 5-year overall survival (OS); and 5-year cancer-specific survival (CSS). These are CORMAC^17^ core outcome measures. Time-to-events were from the date of start of first treatment. LRF was defined as the presence of either residual or recurrent disease within the inguinal/pelvic anatomic sites. OS was defined as the period of time until death from any cause; CSS was defined as the period of time until death from anal cancer.

### Statistical analysis

All statistical analyses were performed using Stata software, Version 14 (Stata Corp, College Station, TX, USA). The main analysis was based on patients receiving curative CRT; sensitivity analyses included all patients treated with curative intent—namely CRT and RT, over the time period. In order to test for a period effect, we divided the cohort into five groups of five-year intervals, spanning the 25-year study period. Differences in baseline characteristics across the five periods were explored using the Cuzick’s non-parametric test and the Cochran-Armitage test for trends (2 × n tables) as appropriate. For cT stage, we used ordinal regression to account for the multinomial stage proportions and examine whether overall stage distribution and stage-specific proportions changed significantly.

To derive predicted contemporary (2020) estimates, we used a two-stage approach. First, we assessed for key confounders in this cohort and evaluated the associations between patient and tumour factors with the three outcomes of 3-year LRF; 5-year OS; and 5-year CSS. We derived Kaplan–Meier (K–M) estimates and then performed univariable and multivariable analyses using Cox models, adjusted for year of treatment. Proportionality assumptions were tested using Schoenfeld residuals.

Second, we sought to relate changes in key outcomes with study periods. For this analysis, we estimated the three outcomes using K–M methods, in two-year bands (except the first 3 years, due to small sample size), and related these over time using regression models, weighted for period sample size. Initial exploration revealed that linear models might predict implausible outcomes (for example, greater than 100% survivals). Therefore, non-linear splines were used. A range of cut-off points from years 2000 to 2010 were tested as pivots for each scenario. The optimal cut-point was determined based on three criteria: (i) visual inspection of plots; (ii) lowest AIC (Akaike Information Criteria) value per model; and (iii) clinically plausible coefficients. For example, if LRF rates were declining (negative regression coefficient), we rejected models where the regression coefficient ‘right’ of the cut-point was positive. Once the optimum regression spline model was determined, we used it to predict options to extrapolate estimates with 95% confidence intervals (95% CIs) for 2020. We additionally tested for the presence of competing risk of death bias by visually comparing the predictions for 5-year OS vs. 5-year CSS over time.

Finally, once we established the optimal regression models, we superimposed the equivalent estimates for the three primary outcomes from the six reported trials^15,16,18–21^ of CRT in patients with SCCA, and visually inspected for model fit.

## Results

### Baseline characteristics

**Fig. 1:**
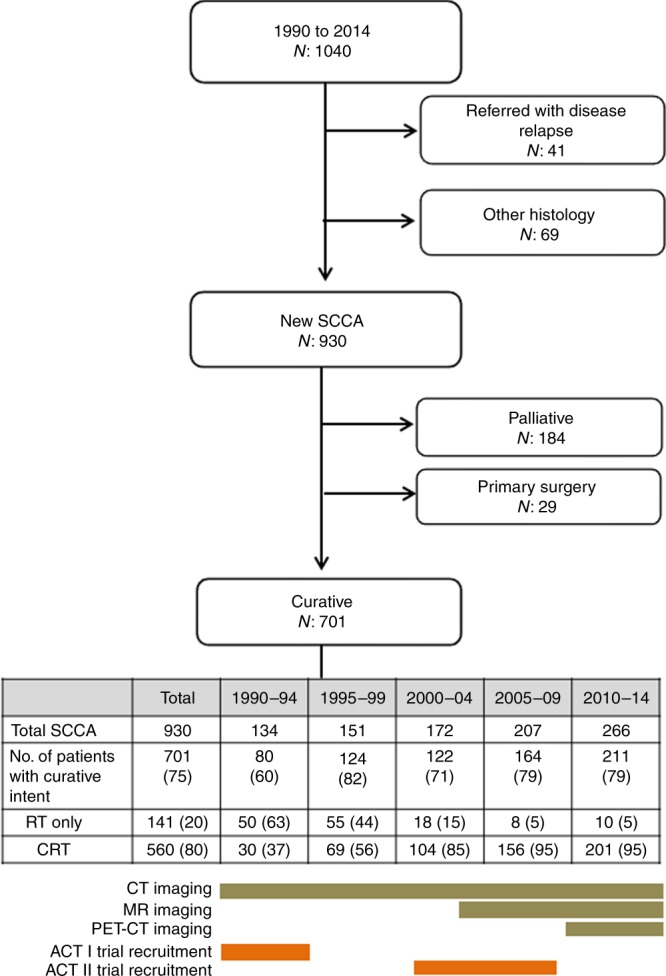
Flow diagram of The Christie squamous cell carcinoma of the anus (SCCA) series, 1990–2014.

Between 1990 and 2014, there were 1040 referrals to the anal cancer MDT of which 930 (89%) were primary SCCA. Numbers of patients with SCCA doubled from 134 in 1990–94 to 266 in 2010–14. Of the 930 patients, there were 701 patients treated with curative intent, with either RT (N: 141) or CRT (N: 560) (Fig. 1). The proportions treated by curative intent remained steady (at approximately 80%) across the five time intervals (lower panel in Fig. 1). Median radiotherapy duration was 37 (IQR: 37–38) days. The proportion of patients with incomplete radiotherapy (<32 days) was 2.7%; the proportion with clinically-relevant delayed delivery of radiotherapy (≥42 days) was 8.0%. There were no differences across time periods from 2005.

Table 1 details the baseline characteristics by time periods for the 560 patients undergoing CRT. Women accounted for two-thirds of the cohort. Median age was 60 years and was stable across the study periods. The proportions of cT1 to cT4 stages remained remarkably stable across the study periods (all *P*s > 0.05). By contrast, nodal positivity increased from 17% in the first study period to 41% in the last study period (*P* < 0.001). The baseline characteristics for the 701 patients undergoing either RT or CRT with curative intent are detailed in Table S1. The proportions and trends with time are very similar to those for CRT alone.

**Table 1:** Baseline characteristics in 560 patients with SCCA treated by chemo-radiotherapy at The Christie 1990–2014, by study period.

	Study periods						
	Total	1990–94	1995–99	2000–04	2005–09	2010–14	*P* value
Number of patients	560	30	69	104	156	201	
Gender							
Women	356 (64)	16 (53)	44 (64)	72 (69)	105 (67)	119 (59)	
Men	204 (36)	14 (47)	25 (36)	32 (31)	51 (33)	82 (41)	0.678^a^
Median age (IQR), years	60 (52–69)	60 (50–65)	57 (51–68)	59 (50–70)	60 (52–69)	61 (53–68)	0.169^b^
Self-reported MSM (% of men)	43 (21)	2 (14)	0	6 (17)	20 (39)	15 (18)	0.110^a^
HIV positivity	18 (3)	0	0	2 (2)	8 (5)	8 (4)	0.006^a^
Performance status (WHO)^c^							
0		–	–	–	51 (60	97 (53)	
1		–	–	–	29 (34)	71 (39)	
2		–	–	–	4 (5)	14 (8)	
3		–	–	–	1	2	0.657
Unknown		–	–	–	71	17	
Anatomic site^d^							
Canal	489 (88)	30 (100)	66 (96)	89 (87)	126 (81)	178 (89)	
Margin	69 (12)	0	3 (4)	13 (13)	30 (19)	23 (11)	0.034^a^
Pre-treatment imaging							
CT scan		30 (100)	69 (100)	103 (99)	149 (96)	193 (96)	0.181
MR scan		NA	1	12 (12)	141 (90)	200 (99)	<0.001
PET-CT scan		NA	NA	NA	5 (3)	129 (64)	<0.001
Histological sub-type							
Squamous cell carcinoma, NOS	505 (90)	24 (80)	60 (87)	90 (86)	135 (87)	196 (98)	
Cloacogenic	10 (2)	1	3 (4)	2 (2)	3 (2)	1	
Basaloid	45 (8)	5 (17)	6 (9)	12 (12)	19 (12)	4 (2)	0.004
Tumour differentiation^d^							
Well	72 (20)	3 (20)	11 (27)	21 (32)	14 (16)	23 (16)	
Moderate	159 (45)	8 (53)	21 (51)	20 (31)	40 (46)	70 (48)	
Poor	123 (35)	4 (27)	9 (22)	24 (37)	33 (38)	53 (36)	0.077
AJCC 7th Ed T stage^d^							
cT1	66 (12)	1	6 (9)	13 (13)	22 (16)	24 (12)	0.137^e^
cT2	91 (45)	14 (47)	25 (37)	43 (42)	57 (40)	91 (45)	base
cT3	45 (22)	7 (23)	18 (26)	29 (28)	37 (26)	45 (22)	0.292^e^
cT4	41 (20)	8 (27)	19 (28)	17 (17)	25 (18)	41 (20)	0.226^e^
Nodal detection^d^							
cN+	157 (30)	5 (17)	10 (15)	20 (21)	39 (29)	83 (41)	<0.001^a^
Pre-treatment colostomy							
Yes	70 (13)	1 (3)	4 (6)	5 (5)	23 (15)	37 (18)	0.001^a^
Median radiotherapy dose, Gy		5550	5500	5040	5040	5040	
Median (IQR) duration of radiotherapy (days)	37 (37–38)	–	–	38 (37–38)	37 (37–38)	37 (37–38)	
Delayed radiotherapy < 32 days duration %	2.7	–	–	0	2.5	3.4	0.602^a^†
Delayed radiotherapy ≥ 42 days duration %	8.0	–	–	4.0	10.5	6.2	0.255^a^†
Chemotherapy agents (% of total chemotherapy)							
Mitomycin C		29 (97)	43 (62)	86 (83)	139 (89)	197 (98)	N/A
iv 5-fluorouracil		30 (100)	68 (99)	87 (84)	146 (94)	201 (38)	N/A
Cisplatin		0	17 (25)	12 (12)	11 (7)	1	N/A
Oral capecitibine		0	0	1	7 (5)	0	N/A

Values in parentheses less otherwise specified. N/A: deemed not appropriate to test trends

AJCC 7th edition: pre-2009 stages recoded accordingly

Prefix ‘c’ indicated pre-treatment clinical staging

*MSM* men who have sex with men, *NOS* not otherwise specified, *CRT* chemoradiotherapy, *IQR* inter-quartile range

^a^Cochran-Armitage test for trends across ordered groups. If not indicated, comparisons across categorical data were chi-squared

^b^Cuzick’s non-parametric test for trends across ordered groups

^c^Performance status was not recorded in the database prior to 2005

^d^Missing data as follows: anatomic site, 2; tumour differentiation, 206; T stage, 18; nodal detection, 29

^e^Ordinal regression

### Locoregional failure

With a median follow-up of 61 (IQR: 36–79) months, there were 119 LRFs among the 560 patients who underwent CRT. The 3-year LRF rate was 33% in the first study period (1990–1994), declining to 16% in the last study period (2010–2014) (*P*_trends_ < 0.001) (Table 2). LFR rates were higher among men compared with women, even after excluding HIV positivity patients (who were all men except one) (*p* = 0.003), and higher with increasing cT stage (cT3 v cT2, *p* = 0.004; cT4 v cT2, *p* < 0.001). Anatomic site, tumour differentiation and nodal positivity were not associated with LRF.

**Table 2:** Cox models for loco-regional failure (LRF) in 560 patients with SCCA treated with chemoradiotherapy, The Christie 1990–2014.

		Univariable	Multivariable		Alternate models/interaction terms
	3-year LRF%	Hazard ratios (95% CIs)	Hazard ratios (95% CIs)	*P* value	
Model A (N: 530)					
Period					
1990–94	33	2.653 (1.337, 5.265)			
1995–99	32	2.438 (1.426, 4.168)			
2000–04	25	1.679 (1.001, 2.819)			
2005–09	13	0.975 (0.574, 1.656)			
2010–14	16	1.000			
Period continuous (per 5 years)	0.757 (0.661, 0.868)	0.776 (0.676, 0.891)	<0.001		
Age category (by median)					
<aged 62 years	20	1.000			
≥aged 62 years	20	1.019 (0.707, 1.468)			
Age continuous (per 5 years)		0.968 (0.895, 1.048)	0.994 (0.918, 1.077)	0.884	
Gender					Adverse effect in men persists after exclusion of HIV+ patients: 361 cases: 1.767^a^ (1.002, 3.114)
Women	17	1.000	1.000	0.003	
Men	25	1.641 (1.139, 2.364)	1.762 (1.215, 2.557)		
Anatomic site					
Canal	20	1.000	1.000	0.466	
Margin	17	0.800 (0.440, 1.454)	1.252 (0.684, 2.294)		
Histological sub-type					
Squamous cell carcinoma, NOS	21	1.000	1.000	0.162	
Cloacogenic	0	Not estimable	0.574^b^ (0.263, 1.249)		
Basaloid	14	0.650 (0.303, 1.396)			
AJCC T stage 7th Ed					No significant interaction term between period and T stage
cT1	2	0.205 (0.049, 0.854)	0.232 (0.055, 0.971)	0.045	
cT2	13	1.000	1.000		
cT3	27	2.097 (1.311, 3.353)	2.033 (1.257, 3.288)	0.004	
cT4	39	3.259 (2.069, 5.133)	3.268 (1.986, 5.278)	<0.001	
Nodal detection					
cN0	18	1.000	1.000	0.530	
cN+	28	1.653 (1.137, 2.405)	1.145 (0.750, 1.748)		
Model B (N: 334)					
Tumour differentiation					
Well	20	0.695 (0.374, 1.293)	0.762 (0.394, 1.472)	0.419	
Moderate	26	1.000	1.000		
Poor	22	0.807 (0.501, 1.300)	0.875 (0.534, 1.432)	0.594	
Models C (*N*: 194)					
Self-reported MSM^c^					
No	26	1.000	1.000	0.952	
Yes	22	0.720 (0.358, 1.448)	0.978 (0.471, 2.031)		
Model D (N: 350)					
HIV positivity^a^					
No	15	1.000	1.000	0.747	
Yes	40	3.102 (1.411, 6.818)	1.162 (0.466, 2.898)		
Model E (N: 256)					
WHO performance status^d^					
0	10	1.000	1.000		
1	18	1.880 (0.948, 3.731)	1.923 (0.944, 3.917)	0.072	
2	45	6.278 (2.739, 14.384)	5.187 (2.094, 12.850)	<0.001	
3	Sample too small				

Prefix ‘c’ indicated clinical staging

Model A: complete case analysis (hence 530 rather than 560 cases) with adjustment for year, age, sex, anatomic site, histological sub-type, T stage, N stage

Model B: model A plus adjustment for differentiation (high proportion of missingness for differentiation)

Model C: model A plus MSM, modelling limited to men

Model D: model A plus HIV status, modelling limited to periods 2000 to 2014

Model E: model A plus performance status, modelling limited to periods 2005 to 2014

*CI* confidence interval, *MSM* men who have sex with men, *NOS* not otherwise specified

^a^HIV status analysis limited to periods 2000–2014

^b^Cloacogenic and basaloid sub-types combined to avoid the mathematical problems of zero events

^c^Models limited to men

^d^Performance status analyses limited to periods 2005–2014

### Survival

There were 230 deaths among the 560 patients who underwent CRT. The 5-year OS rate was 60% in the first study period (1990–1994), increasing to 76% in the last study period (2010–2014) (*P*_trends_ = 0.001) (Table 3). OS rates declined with increasing cT stage (cT4 v cT2, *p* < 0.001), node positivity (*p* = 0.012), and poorer performance status (WHO PS2 v PS0, *p* = 0.049 in analyses limited from 2005 to 2014). Gender, anatomic site, tumour differentiation and HIV positivity were not associated with OS.

**Table 3:** Cox models for overall and cancer-specific survivals in 560 patients with SCCA treated with chemoradiotherapy, The Christie 1990–2014.

	Overall survival (OS)				Cancer-specific survival (CSS)			
		Univariable	Multivariable			Univariable	Multivariable	
	5-year OS%	Hazard ratios (95% CIs)	Hazard ratios (95% CIs)	*P* value	5-year CSS%	Hazard ratios (95% CIs)	Hazard ratios (95% CIs)	*P* value
Model A (*N*: 530)								
Period								
1990–94	60	1.958 (1.032, 3.713)			62	2.211 (1.122, 4.353)		
1995–99	55	2.085 (1.295, 3.357)			62	2.102 (1.242, 3.557)		
2000–04	69	1.366 (0.860, 2.168)			77	1.249 (0.738, 2.115)		
2005–09	72	1.199 (0.784, 1.836)			79	1.057 (0.649, 1.722)		
2010–14	76	1.000			80	1.000		
Period continuous (per 5 years)	0.838 (0.742, 0.946)	0.808 (0.712, 0.917)	0.001		0.838 (0.742, 0.946)	0.794 (0.691, 0.913)	0.001	
Age category (by median)								
<aged 62 years	73	1.000			78	1.000		
≥aged 62 years	66	1.307 (0.955, 1.789)			74	1.307 (0.955, 1.789)		
Age continuous (per 5 years)		1.047 (0.978, 1.121)	1.065 (0.991, 1.144)	0.088		1.034 (0.958, 1.117)	1.042 (0.961, 1.129)	0.319
Gender								
Women	71	1.000	1.000	0.747	76	1.000	1.000	0.759
Men	70	1.065 (0.770, 1.474)	1.058 (0.750, 1.494)		75	1.090 (0.775, 1.571)	1.062 (0.723, 1.560)	
Anatomic site								
Canal	69	1.000	1.000	0.256	75	1.000	1.000	0.340
Margin	81	0.542 (0.301, 0.978)	0.687 (0.60, 1.312)		86	0.520 (0.263, 1.026)	0.703 (0.341, 1.449)	
Histological sub-type								
Squamous cell carcinoma, NOS	70	1.000	1.000		75	1.000	1.000	
Cloacogenic	78	0.728 (0.180, 2.941)	1.030 (0.250, 4.236)	0.967	89	0.463 (0.065, 3.318)	0.636 (0.087, 4.631)	0.655
Basaloid	75	0.806 (0.437, 1.489)	0.802 (0.417, 1.543)	0.509	81	0.743 (0.363, 1.521)	0.670 (0.322, 1.523)	0.368
AJCC T stage 7th Ed								
cT1	91	0.361 (0.144, 0.906)	0.370 (0.133, 1.033)	0.058	97	0.188 (0.045, 0.778)	0.245 (0.058, 1.021)	0.054
cT2	77	1.000	1.000		82	1.000	1.000	
cT3	66	1.638 (1.085, 2.472)	1.406 (0.916, 2.156)	0.119	74	1.626 (1.017, 2.600)	1.417 (0.872, 2.301)	0.159
cT4	50	2.819 (1.906, 4.167)	2.272 (1.482, 3.481)	<0.001	55	3.150 (2.042, 4.858)	2.500 (1.557, 4.015)	<0.001
Nodal detection								
cN0	76	1.000	1.000	0.012	81	1.000	1.000	0.024
cN+	57	2.060 (1.482, 2.864)	1.612 (1.109, 2.344)		64	2.111 (1.460, 3.055)	1.612 (1.064, 2.442)	
Model B (*N*: 334)								
Tumour differentiation								
Well	79	0.683 (0.374, 1.243)	0.718 (0.362, 1.427)	0.345	83	0.673 (0.343, 1.323)	0.794 (0.382, 1.650)	0.537
Moderate	70	1.000	1.000		75	1.000	1.000	
Poor	66	1.141 (0.743, 1.752)	1.166 (0.740, 1.837)	0.509	71	1.170 (0.727, 1.884)	1.195 (0.724, 1.973)	0.486
Models C (*N*: 194)								
Self-reported MSM^a^								
No	71	1.000	1.000	0.204	76	1.000	1.000	0.168
Yes	65	0.720 (0.358, 1.448)	1.508 (0.800, 2.844)		70	1.319 (0.705, 2.467)	1.624 (0.814, 3.238)	
Model D (*N*: 350)								
HIV positivity^b^								
No	74	1.000	1.000	0.196	80	1.000	1.000	0.219
Yes	51	2.320 (1.123, 4.792)	1.759 (0.747, 4.142)		58	2.624 (1.203, 5.725)	1.792 (0.706, 4.544)	
Model E (*N*: 256)								
WHO performance status^c^								
0	79	1.000	1.000		84	1.000	1.000	
1	69	1.672 (0.990, 2.824)	1.761 (0.997, 3.108)	0.051	75	1.876 (1.021, 3.445)	1.790 (0.933, 3.432)	0.080
2	60	2.463 (1.071, 5.659)	2.460 (1.004, 6.032)	0.049	64	2.953 (1.178, 7.400)	2.835 (1.059, 7.589)	0.038
3	Sample too small							

Prefix ‘c’ indicated clinical staging

Model A: complete case analysis (hence 530 rather than 560 cases) with adjustment for year, age, sex, anatomic site, histological sub-type, T stage, N stage

Model B: model A plus adjustment for differentiation (high proportion of missingness for differentiation)

Model C: model A plus MSM, modelling limited to men

Model D: model A plus HIV status, modelling limited to periods 2000 to 2014

Model E: model A plus performance status, modelling limited to periods 2005 to 2014

*CI* confidence interval, *MSM* men who have sex with men, *NOS* not otherwise specified

^a^Models limited to men

^b^HIV status analysis limited to periods 2000–2014

^c^Performance status analyses limited to periods 2005–2014

There were 143 deaths from anal cancer. The 5-year CCS rate was 62% in the first study period (1990–1994), increasing to 80% in the last study period (2010–2014) (*P*_trends_ = 0.001) (Table 3). CSS rates declined with increasing cT stage (cT4 v cT2, *p* < 0.001), node positivity (*p* = 0.024), and poorer performance status (WHO PS2 v PS0, *P* = 0.038; analysis limited to post-2005). Gender, anatomic site, tumour differentiation and HIV positivity were not associated with CSS.

### Modelled estimates

**Fig. 2:**
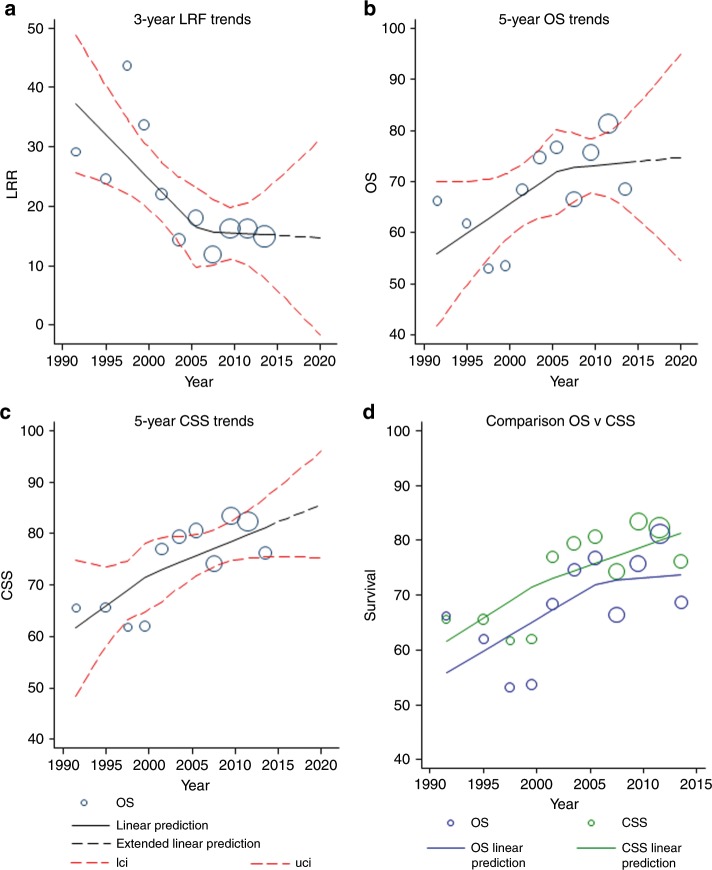
Regression models, with predictions to 2020, of 3-year LRF, 5-year OS and 5-year CSS, and comparative evaluation of OS vs. CSS trends. **a** For 3-year LRF, rates declined from a mean 37.3% in 1991 to 15.6% at the 2007 pivot, and then the decline slowed down to a mean 15.2% in 2014. **b** For 5-year OS, rates increased from a mean 55.9% in 1991 to 72.7% at the 2007 pivot, and then the improvement slowed down to a mean 73.7% in 2014; and **c** for 5-year CSS, rates increased from a mean 61.6% in 1991 to 73.0% at the 2000 pivot, and then the improvement slowed down to a mean 81.3% in 2014. **d** Similar trends for OS and CSS, not supporting a competitive risk bias.

The spline regression models predicted estimates for 3-year LRF, 5-year OS and 5-year CSS similar to those in the observed data but added a non-linear dimension (Fig. 2a–c). For 2020, the predicted 3-year LRF was 14.7% (95% CIs: 0–31.3); 5-year OS was 74.7% (95% CIs: 54.6–94.9); and 5-year CSS was 85.7% (95% CIs: 75.3–96.0). We compared modelled changes with time for 5-year OS and 5-year CSS and found no evidence of a competing risk bias for death (Fig. 2d).

### Predicted models and literature trials

**Fig. 3:**
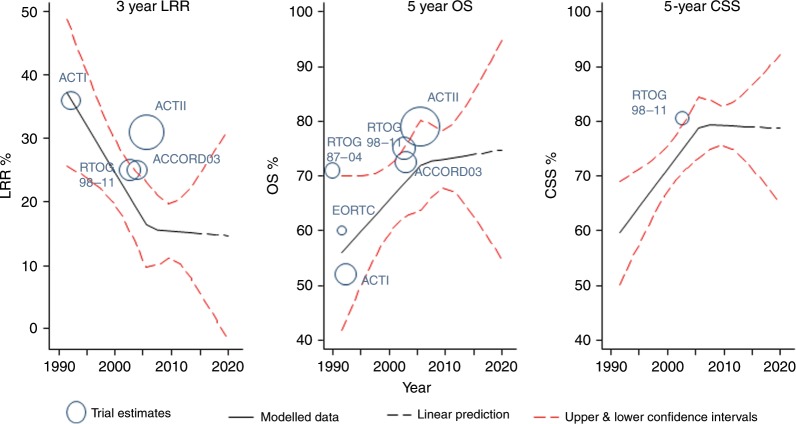
Regression models, with predictions to 2020, of 3-year LRF (loco-regional failure), 5-year OS (overall survival) and 5-year CSS (cancer-specific survival) using splines, as in Fig. 1. The equivalent estimates (either reported or derived indirectly) from each of the published six trials of chemo-radiotherapy in patients with SCCA in superimposed.

We superimposed the equivalent estimates for the three primary outcomes from six published trials of CRT in patients with SCCA. The plots (Fig. 3 and Table S2) illustrate that the current and predicted rates for 3-year LRF and 5-year OS and CSS are considerably improved compared with most of the estimates from historic trials.

### Sensitivity analysis

We repeated the univariable and multivariable models to include all patients treated with curative intent—namely CRT and RT, over the time period, and found similar results (Table S3 and S4).

## Discussion

### Summary of main findings

Over 25 years, we observed the following. First, there were increased numbers of referrals with time and changing treatment selection to predominantly CRT. Second, in the absence of clear evidence of earlier clinical presentation or changing demographics, we illustrated striking improvements in LRF, and OS and CSS with time. Third, we derived models to estimate contemporary oncological outcomes.

### Context of other literature

The increase in number of referrals received by our institute over the 25 years is in keeping with the epidemiological literature, which demonstrates an overall increase in the incidence of anal cancer in many Western populations.^22^

A small number of institute-level studies have described the presentation and outcomes of anal cancer over time. Myerson et al. (Washington University),^23^ Kim et al. (Hwasun Hospital, Korea),^24^ and Tomaszewski et al. (Peter MacCallum Cancer Centre, Australia)^25^ present institutional series over 25–30 years. However, the treatments within the cohorts from Myerson^23^ and Kim^24^ were heterogeneous, and although Tomaszewski et al.^25^ concentrated on patients treated with CRT, this study did not account for the potential effect of time on presentation and outcome. Whitford et al. (Oregon Health Sciences University)^26^ explored for a time period effect on outcome but the study was too small (76 cases over 30 years) to take account of varying presentations with time. Recently, Guren et al.^27^ reported that 5-year net (or relative) survival in 1548 patients from the Cancer Registry of Norway increased from 63 to 73% (1987–2016). However, while the registry reported that 82% were treated with curative intent, detailed treatment details were lacking. Furthermore, relative survival represents a modelled survival estimate taking account underlying period changes, and does not equal observed patient survival estimates, which is required by patients and trialists.

In the 1990s, two randomised trials^15,19^ demonstrated the use of CRT improved local control compared with RT alone. Further trials,^16,20,21^ reported between 2008 and 2013, established the combination of radiotherapy with 5-fluorouracil and mitomycin-C as the optimal therapy. While the use of CRT is associated with improved loco-regional disease control (compared with RT alone), it is unclear whether this translates into improvements in overall survival (argument expanded in Supplemental Material p13). To the best of our knowledge, our study is the first to illustrate parallel temporal improvement for LRF and survivals.

In our analysis, there were striking increases in the proportions of pre-treatment nodal positivity from 17% in 1990–94 to 41% in 2010–14. We believe that most of this is driven by the introduction into clinical practice of modern imaging modalities, a type of the Will-Rogers phenomenon. We have written extensively about this and described the added phenomenon of ‘reduced prognostic discrimination’.^11^ For example, this might explain why nodal positivity was not a predictor of loco-regional relapse. We caution against the interpretation that the increased proportion of nodal positivity reflects a ‘true’ shift to more advanced stage disease, as the proportions of T stages remained constant over the study period.

### Limitations and strengths

Our study has limitations. First, there may be selection bias. Over the study period, improvements might reflect stricter criteria for curative intent. This seems unlikely as the proportions treated by curative intent were broadly 80% throughout. Similarly, improvements might reflect proportionately increased use of CRT (rather than RT). This is true—though our sensitivity analyses demonstrate that the same patterns of oncological outcomes were seen for the combined RT and CRT cohort. Second, there may be unmeasured confounding. For example, we did not routinely capture performance status data before 2005. It is likely that our patients’ general health status improved with time, though it seems less plausible that this alone accounts for the observed 16% absolute improvement in overall survival. Third, there was a lack of treatment-related toxicity data. It is conceivable that grade 3 and 4 toxicities lessened, and their management improved. Again, it seems unlikely that this alone accounted for the magnitude of observed improvements in overall survival. Fourth, we did not capture technical refinements in salvage surgery over time, which might account for some increases in long-term disease-free states. However, as primary locoregional failure rates have reduced substantially, salvage surgery is now less often required. Furthermore, among patients with local relapses, the proportion that proceed to salvage surgery has decreased from more than 70% in historic series^12,28^ in the 1990s to only 23% in the ACT II trial from the mid-2000s.^29^ This is an area of ongoing research in this cohort.

There are several study strengths. First, we used a prospectively maintained database, where for example, key prognostic factors such as pre-treatment stage were consistently recorded. Second, this is the largest temporal clinical institute-level dataset of its type. Other datasets (106 patients;^23^ 50 patients;^24^ 284 patients;^25^ 76 patients^26^)—were smaller. Third, we concentrated our analysis on a homogenous treatment—namely CRT. The details of this treatment varied with time, but the backbone was 50–55 Gy radiotherapy and a 5-FU-mitomycin based concurrent chemotherapy. Fourth, there was appropriate length follow-up. Fifth, our definition of 3-year LRF is equivalent to that currently used in the UK PLATO trial,^30^ and many of the patient population in the primary analysis of this study are equivalent to those eligible for modern trials, like PLATO.

### Clinical implications

The improved oncological outcomes are likely to have multifactorial drivers. The use of advanced imaging may facilitate more accurate treatment with CRT. Advances in RT technologies over time, better awareness of toxicity and improved supportive care and the abandonment of the inter-phase RT break (after ACT I) are likely contributors. Centralisation of anal cancer management is likely to have contributed to improvements through use of defined patient protocols. The culmination of these changes is the probable driver of improved oncological outcomes, although near-impossible to quantify. Human Papilloma Virus (HPV) is the aetiological agent in most SCCA tumours, but is also a marker of radio-sensitivity.^31^ It is conceivable that the proportion of HPV-driven tumours have increased with time, in turn, increasing the overall radio-sensitivity of these cancers.

The current and predicted rates for 3-year LRF and 5-year survivals are more optimistic than those in the historic trials. It is important that ongoing and future trials are appropriately powered to reflect event rates for current standard of care (the control arm). We illustrate this as follows. Consider a hypothetical trial based on clinical practice 25 years ago. We assume that the LRF rate was 30% and the new intervention aimed to improve LRF by (relative) 25% i.e. to 24%. Assuming an alpha = 0.05 and power = 0.80, a 1:1 head-to-head trial would require 675 in each arm (total: 1350) with 365 events. Now consider a similar trial today. We assume that the LRF rate is 20% and the new intervention aimed to improve LRF by (relative) 25% i.e. to 15%. Assuming an alpha = 0.05 and power = 0.80, a 1:1 head-to-head trial would require 715 in each arm (total: 1430) with 251 events.

### Unanswered questions and future research

First, while the Will Rogers phenomenon^11^ may partly explained the increase in proportions of SCCA patients with node positivity, there might be other factors. Nodal positivity is clinically important as this is used as treatment stratification in clinical practice and in trials. The relevance of this is still not clear.

Second, we are now in an era of accurate radiotherapy delivery with VMAT and IMRT, which has become standard RT for anal cancer.^32^ RTOG 0529^33^ was a phase 2 evaluation of dose-painted intensity modulated radiation therapy in combination with 5-FU and MMC, which not only showed a reduction of acute morbidity but also improved LRF. If there is a causal relationship between LRF and OS, these new treatment modalities might further improve LRF, reduce treatment-related toxicity, and ultimately, further reduce the death burden from this cancer.

## Supplementary information

Supplemental material

## Data Availability

This study used routinely collected hospital data kept at The Christie NHS Foundation Trust. The approvals initially received for this study did not allow for the sharing of data, so the data used for the analysis in this study is not currently available.
