# Supplementary material for: Temporal improvements in loco-regional failure and survival in patients with anal cancer treated with chemo-radiotherapy: treatment cohort study (1990–2014)
Source: Br J Cancer. 2020 Jan 14;122(6):749–58. doi: 10.1038/s41416-019-0689-x (PMC7078229; doi:10.1038/s41416-019-0689-x)
Supplement: Supplementary file 1 — Supplemental material [file 41416_2019_689_MOESM1_ESM.docx]

**Electronic Webappendix**

**Temporal improvements in loco-regional failure and survival in patients with anal cancer treated with chemo-radiotherapy: treatment cohort study (1990-2014)**

**Sekhar et al.**

**Table S1 Baseline characteristics in 701 patients with SCCA treated by radiotherapy only and chemoradiotherapy at the Christie 1990 to 2014 by study period**

|  | **Study periods** | | | | | | |
| --- | --- | --- | --- | --- | --- | --- | --- |
|  | **Total** | **1990-94** | **1995-99** | **2000-04** | **2005-09** | **2010-14** | **P value** |
| **Treatment modality** |  |  |  |  |  |  |  |
| RT only | 141 (20) | 50 (63) | 55 (44) | 18 (15) | 8 (5) | 10 (5) |  |
| CRT | 560 (80) | 30 (37) | 69 (56) | 104 (85) | 156 (95) | 201 (95) | < 0.001† |
| **Number of patients** | 701 | 80 | 124 | 122 | 164 | 211 |  |
| **Median age (range), years** | 62  (29-92) | 63  (37-83) | 64  (33-91) | 62  (36-92) | 61  (29-87) | 61  (29-91) | 0.362* |
| **Gender** |  |  |  |  |  |  |  |
| Women | 450 (64) | 46 (58) | 82 (66) | 84 (69) | 112 (68) | 126 (60) |  |
| Men | 251 (36) | 34 (42) | 42 (34) | 38 (31) | 52 (32) | 85 (40) | 0.825† |
| **Self-reported MSM (% of men)** | 46 (18) | 5 (15) | 0 | 6 (15) | 20 (38) | 15 (17) | 0.027† |
| **HIV positivity** | 18 (3) | 0 | 0 | 2 (2) | 8 (5) | 8 (4) | 0.006† |
| **Performance status (WHO) ¶¶** |  |  |  |  |  |  |  |
| 0 |  | - | - | - | 51 (58) | 98 (51) |  |
| 1 |  | - | - | - | 31 (35) | 75 (39) |  |
| 2 |  | - | - | - | 4 (5) | 17 (9) |  |
| 3 |  | - | - | - | 2 (2) | 4 (2) | 0.516 |
| Unknown |  |  |  |  | 76 | 17 |  |
| **Anatomic site¶** |  |  |  |  |  |  |  |
| Canal | 611 (87) | 73 (91) | 116 (94) | 106 (88) | 130 (80) | 186 (88) |  |
| Margin | 87 (13) | 7 (9) | 8 (6) | 14 (12) | 33 (20) | 25 (12) | 0.051† |
| **Pre-treatment imaging** |  |  |  |  |  |  |  |
| CT scan |  | 80 (100) | 124 (100) | 120 (98) | 157 (96) | 203 (96) |  |
| MR scan |  | 0 | 1 | 13 (11) | 145 (88) | 209 (99) |  |
| PET-CT scan |  | 0 | 0 | 0 | 5 (3) | 137 (65) |  |
| **Histological sub-type** |  |  |  |  |  |  |  |
| Squamous cell carcinoma, NOS | 628 (90) | 65 (81) | 113 (91) | 104 (85) | 141 (86) | 205 (97) |  |
| Cloacogenic | 13 (2) | 2 (3) | 3 (2) | 4 (3) | 3 (2) | 1 (1) |  |
| Basaloid | 60 (8) | 13 (16) | 8 (7) | 14 (12) | 20 (12) | 5 (2) | 0.001 |
| **Tumour differentiation¶** |  |  |  |  |  |  |  |
| Well | 89 (20) | 10 (21) | 18 (24) | 22 (31) | 15 (16) | 24 (16) |  |
| Moderate | 203 (46) | 26 (55) | 38 (50) | 24 (33) | 42 (46) | 73 (47) |  |
| Poor | 148 (34) | 11 (24) | 20 (26) | 26 (36) | 34 (38) | 57 (37) | 0.083 |
| **Pre-treatment colostomy** |  |  |  |  |  |  |  |
| Yes | 75 (11) | 2 (3) | 5 (4) | 7 (6) | 23 (14) | 38 (18) | < 0.001† |
| **AJCC 7^th^ Ed T stage¶** |  |  |  |  |  |  |  |
| cT1 | 81 (12) | 4 (5) | 12 (10) | 16 (13) | 24 (17) | 25 (12) | 0.137‡ |
| cT2 | 292 (43) | 36 (45) | 48 (39) | 51 (43) | 59 (41) | 98 (47) | base |
| cT3 | 167 (25) | 21 (26) | 31 (25) | 33 (27) | 37 (25) | 45 (21) | 0.292‡ |
| cT4 | 139 (20) | 19 (24) | 32 (26) | 20 (17) | 25 (17) | 43 (20) | 0.226‡ |
| **Nodal Detection¶** |  |  |  |  |  |  |  |
| cN+ | 168 (25) | 5 (6) | 14 (11) | 22 (20) | 40 (29) | 87 (41) | < 0.001† |
| **Median radiotherapy dose, Gy** |  |  |  |  |  |  |  |
| RT only |  | 55.0 | 55.0 | 50.4 | 50.4 | 50.4 |  |
| CRT |  | 55.0 | 55.0 | 50.4 | 50.4 | 50.4 | N/A |
| **Chemotherapy agents**  **(% of total chemotherapy)** |  |  |  |  |  |  |  |
| Mitomycin C | 494 (88) | 29 (97) | 43 (62) | 86 (83) | 139 (89) | 197 (98) | N/A |
| iv 5-fluorouracil | 542 (95) | 30 (100) | 68 (99) | 87 (84) | 146 (94) | 201 (100) | N/A |
| Cisplatin | 41 (7) | 0 | 17 (25) | 12 (12) | 11 (7) | 1 (1) | N/A |
| Oral capecitibine | 8 (1) | 0 | 0 | 1 (1) | 7 (4) | 0 | N/A |
|  |  |  |  |  |  |  |  |

Values in parentheses less otherwise specified. N/A: deemed not appropriate to test for trends

MSM: men who have sex with men. NOS: not otherwise specified. RT: radiotherapy. CRT chemoradiotherapy. Prefix ‘c’ indicated pre-treatment clinical staging.

* Cuzick’s non-parametric test for trends across ordered groups

† Cochran-Armitage test for trends across ordered groups. If not indicated, comparisons across categorical data were chi-squared.

‡ Multi-nominal logistic regression.

¶ Missing data as follows: anatomic site, 3; tumour differentiation, 261; T stage, 22; nodal detection, 35.

¶¶ Performance status was not recorded in the database prior to period 4.

**Table S2 Lack of influence of local control on survival – from six published randomised trials**

| **Study** | **Arm** | **Treatment description** | **No.** | **Loco-regional failure** | **Cancer specific survival** | **Overall survival** |
| --- | --- | --- | --- | --- | --- | --- |
| ACTI 1996 [[1](#_ENREF_1)] | Control | Radiotherapy alone | 279 | local failure: 59% |  | 5-year OS: 52%*  HR: 0.86 (0.67-1.11) |
|  | Experimental | Chemoradiotherapy (5FU + Mitomycin) | 283 | local failure: 36% |  |  |
| RTOG 87-04 1996 [[2](#_ENREF_2)] | Control | 5-FU plus rdaiotherapy | 145 | Positive post-treatment biopsy: 15% |  | Crude death rates: 29% vs. 22%; P = 0.31 |
|  | Experimental | 5-FU, mitomycin plus radiothearpy | 146 | Positive post-treatment biopsy: 7% |  |  |
| EORTC 1997 [[3](#_ENREF_3)] | Control | Radiotherapy alone | 55 | complete response: 54% |  | 5-year OS: 60%*  P = 0.17 |
|  | Experimental | Chemoradiotherapy (5FU + Mitomycin) | 55 | complete response: 85% |  |  |
| RTOG 98-11 2008 [[4](#_ENREF_4)] | Control | 5-FU plus mitomycin and radiotherapy | 324 | 5-year locoregional recurrence rate: 25% |  | 5-year OS: 75% |
|  | Experimental | 5-FU plus cisplatin and radiotherapy | 320 | 5-year locoregional recurrence rate: 33% |  | 5-year OS: 70% |
| ACCORD 2012 [[5](#_ENREF_5)] | Arm A | Two ICT cycles, pelvic RCT, and standard-dose (SD) boost | 75 | 5-year local control: 72.0% | 5-year CSS: 76.6 | 5-year OS: 72.5%† |
|  | Arm B | Two ICT cycles, pelvic RCT, and high-dose (HD) boost. | 75 | 5-year local control: 87.6% | 5-year CSS: 88.8 |  |
|  | Arm C | Neoadjuvant chemo, RCT and SD boost | 82 | 5-year local control: 83.7% | 5-year CSS: 80.6 |  |
|  | Arm D | Neoadjuvant chemo, RCT and HD boost | 75 | 5-year local control: 78.0% | 5-year CSS: 75.9 |  |
| ACTII 2013 [[6](#_ENREF_6)] | Control | Mitomycin-based chemotherapy plus RT | 472 | complete response: 90.5% |  | 5-year OS: 79% |
|  | Experimental | Cisplatin-based chemotherapy plus RT | 468 | complete response: 89.6% |  | 5-year OS: 77% |

* Estimate from K-M curve

† ‘Average from 5-year survivals for each of the four arms

**Table S3 Multivariable Cox models for loco-regional failure (LRF) in 701 patients with SCCA treated with radiotherapy alone or chemoradiotherapy, Christie 1990 to 2014**

|  |  | Univariable | Multivariable | | Alternate models/ interaction terms |
| --- | --- | --- | --- | --- | --- |
|  | 3-year LRF% | Hazard ratios (95% CIs) | Hazard ratios (95% CIs) | P value |  |
|  |  |  | **Model A (N: 665)** | |  |
| **Treatment** | |  |  |  |  |
| RT only | 51 | 1.000 | 1.000 |  |  |
| CRT | 20 | 0.311  (0.230, 0.419) | 0.423  (0.284, 0.628) | < 0.001 |  |
| **Period** |  |  |  |  |  |
| 1990-94 | 49 | 3.528  (2.249, 5.534) |  |  |  |
| 1995-99 | 39 | 2.857  (1.867, 4.374) |  |  |  |
| 2000-04 | 28 | 1.688  (1.056, 2.699) |  |  |  |
| 2005-09 | 13 | 0.899  (0.545, 1.486) |  |  |  |
| 2010-14 | 18 | 1.000 |  |  |  |
| **Period continuous**  **(per 5 years)** | | 0.707  (0.639, 0.780) | 0.826  (0.728, 0.937) | 0.003 |  |
| **Age category (by median)** | |  |  |  |  |
| < aged 62 years | 21 | 1.000 |  |  |  |
| ≥ aged 62 years | 31 | 1.611  (1.201, 2.163) |  |  |  |
| **Age continuous**  **(per 5 years)** |  | 1.094  (1.030, 1.161) | 1.083  (1.019, 1.152) | 0.011 |  |
| **Gender** |  |  |  |  | Adverse effect in men persists after exclusion of HIV+ patients: 348 cases:  1.934†  (1.012, 3.326) |
| Women | 24 | 1.000 | 1.000 | 0.009 |  |
| Men | 30 | 1.382  (1.032, 1.852) | 1.483  (1.105, 1.998) |  |  |
| **Anatomic site** |  |  |  |  |  |
| Canal | 27 | 1.000 | 1.000 | 0.230 |  |
| Margin | 23 | 0.8423  (0.529, 1.340) | 1.334  (0.832, 2.150) |  |  |
| **Histological sub-type** | |  |  |  |  |
| Squamous cell carcinoma, NOS | 26 | 1.000 | 1.000 | 0.986 |  |
| Cloacogenic | 18 | 0.552  (0.137, 2.224) | 0.996¶  (0.611, 1.622) |  |  |
| Basaloid | 28 | 1.005  (0.609, 1.654) |  |  |  |
| **AJCC T stage 7^th^ Ed** | |  |  |  | No significant interaction term between period and T stage |
| cT1 | 5 | 0.263  (0.106, 0.655) | 0.303  (0.121, 0.757) | 0.011 |  |
| cT2 | 22 | 1.000 |  |  |  |
| cT3 | 33 | 1.641  (1.146, 2.349) | 1.724  (1.191, 2.498) | 0.004 |  |
| cT4 | 43 | 2.307  (1.621, 3.284) | 2.389  (1.643, 3.476) | < 0.001 |  |
| **Nodal Detection** |  |  |  |  |  |
| cN0 | 26 | 1.000 | 1.000 | 0.161 |  |
| cN+ | 31 | 1.193  (0.865, 1.643) | 1.302  (0.901, 1.881) |  |  |
|  |  |  | **Model B (N: 416)** | |  |
| **Tumour differentiation** | |  |  |  |  |
| Well | 22 | 0.552  (0.334, 0.914) | 0.466  (0.266, 0.815) | 0.007 |  |
| Moderate | 36 | 1.000 | 1.000 |  |  |
| Poor | 19 | 0.754  (0.518, 1.094) | 0.821  (0.559, 1.205) | 0.594 |  |
|  |  |  | **Models C (N: 241)** | |  |
| **Self-reported MSM*** | |  |  |  |  |
| No | 31 | 1.000 | 1.000 | 0.864 |  |
| Yes | 23 | 0.713  (0.394, 1.289) | 1.055  (0.569, 1.966) |  |  |
|  |  |  | **Model D (N: 366)** | |  |
| **HIV positivity†** |  |  |  |  |  |
| No | 17 | 1.000 | 1.000 | 0.829 |  |
| Yes | 40 | 2.812  (1.287, 6.144) | 1.103  (0.449, 2.708) |  |  |
|  |  |  | **Model E (N: 265)** | |  |
| **WHO performance status ‡** | |  |  |  |  |
| 0 | 10 | 1.000 | 1.000 |  |  |
| 1 | 19 | 2.013  (1.031, 3.933) | 2.141  (1.067, 4.299) | 0.032 |  |
| 2 | 49 | 6.739  (3.088, 14.71) | 5.448  (2.287, 12.97) | < 0.001 |  |
| 3 | Sample too small |  |  |  |  |

CI: confidence interval. RT: radiotherapy. CRT: chemoradiotherapy. MSM: men who have sex with men. NOS: not otherwise specified. Prefix ‘c’ indicated clinical staging.

*Models limited to men.

† HIV status analysis limited to periods 2000 to 2014.

‡ Performance status analyses limited to periods 2005 to 2014.

¶ Cloacogenic and basaloid sub-types combined to avoid the mathematical problems of zero events.

Model A: complete case analysis (hence 665 rather than 701 cases) with adjustment for treatment, year, age, sex, anatomic site, histological sub-type, T stage, N stage.

Model B: model A plus adjustment for differentiation (high proportion of missingness for differentiation)

Model C: model A plus MSM, modelling limited to men

Model D: model A plus HIV status, modelling limited to periods 2000 to 2014

Model E: model A plus performance status, modelling limited to periods 2005 to 2014

**Table S4 Cox models for overall and cancer-specific survivals in 701 patients with SCCA treated with radiotherapy only and chemoradiotherapy, Christie 1990 to 2014**

|  | **Overall survival (OS)** | | | | **Cancer-specific survival (CSS)** | | | |
| --- | --- | --- | --- | --- | --- | --- | --- | --- |
|  |  | Univariable | Multivariable |  |  | Univariable | Multivariable |  |
|  | 5-year OS% | Hazard ratios (95% CIs) | Hazard ratios (95% CIs) | P value | 5-year CSS% | Hazard ratios (95% CIs) | Hazard ratios (95% CIs) | P value |
|  |  |  | **Model A (N: 665)** | |  |  | **Model A (N: 665)** | |
| **Treatment** |  |  |  |  |  |  |  |  |
| RT only | 51 | 1.000 | 1.000 |  | 58 | 1.000 | 1.000 |  |
| CRT | 70 | 0.477  (0.353, 0.645) | 0.554  (0.371, 0.828) | 0.004 | 76 | 0.463  (0.331, 0.647) | 0.561  (0.359, 0.876) | 0.011 |
| **Period** |  |  |  |  |  |  |  |  |
| 1990-94 | 56 | 1.898  (1.229, 2.929) |  |  | 60 | 2.139  (1.333, 3.435) |  |  |
| 1995-99 | 53 | 1.975  (1.331, 2.931) |  |  | 60 | 2.090  (1.348, 3.241) |  |  |
| 2000-04 | 68 | 1.266  (0.830, 1.933) |  |  | 76 | 1.184  (0.726, 1.929) |  |  |
| 2005-09 | 71 | 1.074  (0.719, 1.604) |  |  | 78 | 1.009  (0.636, 1.600) |  |  |
| 2010-14 | 74 | 1.000 |  |  | 79 | 1.000 |  |  |
| **Period continuous**  **(per 5 years)** | | 0.840  (0.765, 0.921) | 0.896  (0.794, 1.010) | 0.073 |  | 0.810  (0.730, 0.899) | 0.862  (0.755, 0.985) | 0.029 |
| **Age category (by median)** | |  |  |  |  |  |  |  |
| < aged 62 years | 74 | 1.000 |  |  | 79 | 1.000 |  |  |
| ≥ aged 62 years | 59 | 1.772  (1.345, 2.331) |  |  | 66 | 1.707  (1.254, 2.323) |  |  |
| **Age continuous**  **(per 5 years)** |  | 1.128  (1.067, 1.193) | 1.121  (1.056, 1.191) | <0.001 |  | 1.119  (1.051, 1.192) | 1.107  (1.036, 1.183) | 0.003 |
| **Gender** |  |  |  |  |  |  |  |  |
| Women | 67 | 1.000 | 1.000 | 0.341 | 73 | 1.000 | 1.000 | 0.599 |
| Men | 66 | 1.105  (0.838, 1.457) | 1.151  (0.861, 1.539) |  | 72 | 1.073  (0.785, 1.466) | 1.091  (0.788, 1.511) |  |
| **Anatomic site** |  |  |  |  |  |  |  |  |
| Canal | 65 | 1.000 | 1.000 | 0.649 | 72 | 1.000 | 1.000 | 0.841 |
| Margin | 76 | 0.640  (0.399, 1.025) | 0.889  (0.538, 1.471) |  | 81 | 0.641  (0.377, 1.089) | 0.945  (0.542, 1.646) |  |
| **Histological sub-type** | |  |  |  |  |  |  |  |
| Squamous cell carcinoma, NOS | 66 | 1.000 | 1.000 |  | 73 | 1.000 | 1.000 |  |
| Cloacogenic | 76 | 0.778  (0.249, 2.433) | 1.066  (0.337, 3.376) | 0.912 | 84 | 0.659  (0.163, 2.658) | 0.881  (0.216, 3.596) | 0.860 |
| Basaloid | 67 | 0.968  (0.604, 1.550) | 1.002  (0.611, 1.645) | 0.991 | 72 | 0.963  (0.567, 1.637) | 0.956  (0.546, 1.675) | 0.876 |
| **AJCC T stage 7^th^ Ed** | |  |  |  |  |  |  |  |
| cT1 | 86 | 0.462  (0.231, 0.925) | 0.484  (0.231, 1.012) | 0.058 | 94 | 0.253  (0.092, 0.699) | 0.304  (0.110, 0.843) | 0.022 |
| cT2 | 74 | 1.000 | 1.000 |  | 78 | 1.000 | 1.000 |  |
| cT3 | 63 | 1.562  (1.099, 2.219) | 1.482  (1.029, 2.134) | 0.034 | 71 | 1.484  (1.000, 2.203) | 1.394  (0.927, 2.096) | 0.111 |
| cT4 | 45 | 2.676  (1.992, 3.725) | 2.353  (1.649, 3.357) | < 0.001 | 52 | 2.777  (1.931, 3.993) | 2.400  (1.625, 3.545) | < 0.001 |
| **Nodal Detection** |  |  |  |  |  |  |  |  |
| cN0 | 71 | 1.000 | 1.000 | 0.017 | 76 | 1.000 | 1.000 | 0.014 |
| cN+ | 56 | 1.606  (1.197, 2.153) | 1.521  (1.077, 2.146) |  | 63 | 1.658  (1.197, 1.295) | 1.615  (1.103, 2.368) |  |
|  |  |  | **Model B (N: 416)** | |  |  | **Model B (N: 416)** | |
| **Tumour differentiation** | |  |  |  |  |  |  |  |
| Well | 77 | 0.570  (0.339, 0.959) | 0.641  (0.355, 1.159) | 0.141 | 81 | 0.559  (0.317, 0.986) | 0.671  (0.361, 1.248) | 0.208 |
| Moderate | 64 | 1.000 | 1.000 |  | 69 | 1.000 | 1.000 |  |
| Poor | 63 | 0.999  (0.694, 1.439) | 1.008  (0.688, 1.478) | 0.964 | 69 | 0.937  (0.626, 1.402) | 0.947  (0.623, 1.439) | 0.798 |
|  |  |  | **Models C (N: 241)** | |  |  | **Models C (N: 241)** | |
| **Self-reported MSM*** | |  |  |  |  |  |  |  |
| No | 65 | 1.000 | 1.000 | 0.826 | 72 | 1.000 | 1.000 | 0.540 |
| Yes | 68 | 0.858  (0.493, 1.492) | 1.069  (0.588, 1.942) |  | 72 | 0.965  (0.529, 1.760) | 1.230  (0.635, 2.382) |  |
|  |  |  | **Model D (N: 366)** | |  |  | **Model D (N: 366)** | |
| **HIV positivity†** |  |  |  |  |  |  |  |  |
| No | 73 | 1.000 | 1.000 | 0.187 | 79 | 1.000 | 1.000 | 0.228 |
| Yes | 51 | 2.102  (1.022, 4.324) | 1.765  (0.758, 4.109) |  | 58 | 2.418  (1.113, 5.253) | 1.758  (0.703, 4.399) |  |
|  | |  | **Model E (N: 256)** | |  |  | **Model E (N: 265)** | |
| **WHO performance status ‡** | |  |  |  |  |  |  |  |
| 0 | 79 | 1.000 | 1.000 |  | 85 | 1.000 | 1.000 |  |
| 1 | 68 | 1.791  (1.073, 2.989) | 1.761  (0.997, 3.108) | 0.051 | 74 | 1.978  (1.089, 3.592) | 2.022  (1.067, 3.835) | 0.031 |
| 2 | 51 | 3.206  (1.551, 6.627) | 2.460  (1.004, 6.031) | 0.049 | 58 | 3.595  (1.573, 8.217) | 3.013  (1.188, 7.637) | 0.020 |
| 3 | Sample too small |  |  |  |  |  |  |  |

CI: confidence interval. RT: radiotherapy. CRT: chemoradiotherapy.MSM: men who have sex with men. NOS: not otherwise specified. Prefix ‘c’ indicated clinical staging.

*Models limited to men.

† HIV status analysis limited to periods 2000 to 2014.

‡ Performance status analyses limited to periods 2005 to 2014.

Model A: complete case analysis (hence 665 rather than 701 cases) with adjustment for treatment, year, age, sex, anatomic site, histological sub-type, T stage, N stage.

Model B: model A plus adjustment for differentiation (high proportion of missingness for differentiation)

Model C: model A plus MSM, modelling limited to men

Model D: model A plus HIV status, modelling limited to periods 2000 to 2014

Model E: model A plus performance status, modelling limited to periods 2005 to 2014

**Discussion on literature: lack of clear evidence of effect of improved LRF on improved survival**

In the 1990s, two randomized trials [[3](#_ENREF_3), [7](#_ENREF_7)] demonstrated the use of CRT improved local control compared with RT alone. Further trials [[4-6](#_ENREF_4)], reported between 2008 and 2013, established the combination of radiotherapy with 5-fluorouracil (5-FU) and mitomycin-C (MMC) as the optimal therapy. While the use of CRT is associated with improved loco-regional disease control (compared with RT alone), it is unclear whether this translates into improvements in overall survival. Thus, for example, in the ACT I trial, [[7](#_ENREF_7)] crude loco-regional failure (LRF) reduced from 59% to 36% (p < 0.0001), but the hazard ratio for overall survival fell short of statistically significant benefit. Similarly, in the RTOG 98-11 trial, [[4](#_ENREF_4)] 5-year LRF rates were 25% and 33% for the two arms (RT plus 5-FU and MMC versus RT plus 5-FU and cisplatin) but corresponding 5-year overall survival rates (75% and 70%) were not significantly different. The ACCORD trial [[5](#_ENREF_5)] reported on fours arms with respective 5-year local controls of 72.0%, 87.6%, 83.7%, and 78.0%; and while the corresponding 5-year cancer-specific survival rates broadly correlated (76.6%, 88.8%, 80.6%, and 75.9%), there were no statistical significant differences. It is possible that lack of impact of improved local control on survival may reflect underpowered sample sizes.

**Powering current and future trials based on our results**

The PLATO [[8](#_ENREF_8)] trial is a single protocol ‘umbrella platform’ comprising the ACT3, 4 and 5 trials formed from a network of UK and international multi-disciplinary trialists, motivated to determine a stratified approach to the management of anal cancer, across the spectrum from early to locally advanced stage disease. The primary endpoint across all three sub-trials is 3-year LRF. As the overarching aim is to deliver at least equivalent current LRF, but with acceptable levels of toxicity, our findings are relevant here. In the high-risk sub-group, the ACT5 trial (T2 N1-3, T3/4 N any) is powered on detecting an absolute difference of 10% in 3 year actuarial LRF rates (i.e. time to event rates) between the control and preferred experimental arm. This assumes a LRF rate of 30% in the control arm based on the ACT2 data [[6](#_ENREF_6)]. Based on our data, and accepting that ACT5 is high-risk, this might be a pessimistically high estimate for LRF.

We illustrate the potential lack of power issues as follows: Consider a hypothetical trial based on clinical practice 25 years ago. We assume that the LRF rate was 30% and the new intervention aimed to improve LRF by (relative) 25% i.e. to 24%. Assuming an alpha = 0.05 and power = 0.80, a 1:1 head-to-head trial would require 675 in each arm (total: 1350) with 365 events.

Now consider similar trial today. We assume that the LRF rate is 20% and the new intervention aimed to improve LRF by (relative) 25% i.e. to 15%. Assuming an alpha = 0.05 and power = 0.80, a 1:1 head-to-head trial would require 715 in each arm (total: 1430) with 251 events.

**References**

1. Arnott SJ, Cunningham D, Gallagher J et al. Epidermoid anal cancer: Results from the UKCCCR randomised trial of radiotherapy alone versus radiotherapy, 5-fluorouracil, and mitomycin. Lancet 1996; 348: 1049-1054.

2. Flam M, John M, Pajak TF et al. Role of mitomycin in combination with fluorouracil and radiotherapy, and of salvage chemoradiation in the definitive nonsurgical treatment of epidermoid carcinoma of the anal canal: results of a phase III randomized intergroup study. J Clin Oncol 1996; 14: 2527-2539.

3. Bartelink H, Roelofsen F, Eschwege F et al. Concomitant radiotherapy and chemotherapy is superior to radiotherapy alone in the treatment of locally advanced anal cancer: Results of a phase III randomized trial of the European organization for research and treatment of cancer radiotherapy and gastrointestinal cooperative groups. Journal of Clinical Oncology 1997; 15: 2040-2049.

4. Ajani JA, Winter KA, Gunderson LL et al. Fluorouracil, mitomycin, and radiotherapy vs fluorouracil, cisplatin, and radiotherapy for carcinoma of the anal canal: a randomized controlled trial. JAMA 2008; 299: 1914-1921.

5. Peiffert D, Tournier-Rangeard L, Gerard JP et al. Induction chemotherapy and dose intensification of the radiation boost in locally advanced anal canal carcinoma: final analysis of the randomized UNICANCER ACCORD 03 trial. J Clin Oncol 2012; 30: 1941-1948.

6. James RD, Glynne-Jones R, Meadows HM et al. Mitomycin or cisplatin chemoradiation with or without maintenance chemotherapy for treatment of squamous-cell carcinoma of the anus (ACT II): A randomised, phase 3, open-label, 2×2 factorial trial. The Lancet Oncology 2013; 14: 516-524.

7. Northover JMA, Arnott SJ, Cunningham D et al. Epidermoid anal cancer: Results from the UKCCCR randomised trial of radiotherapy alone versus radiotherapy, 5-fluorouracil, and mitomycin. Lancet 1996; 348: 1049-1054.

8. PLATO_trial. PersonaLising Anal cancer radioTherapy dOse – Incorporating ACT3, ACT4 and ACT5 <http://medhealth.leeds.ac.uk/info/430/solid_tumours/2210/plato> [accessed 20 sEPT 2018].
